# Facile synthesis of a ZnO–BiOI p–n nano-heterojunction with excellent visible-light photocatalytic activity

**DOI:** 10.3762/bjnano.9.72

**Published:** 2018-03-05

**Authors:** Mengyuan Zhang, Jiaqian Qin, Pengfei Yu, Bing Zhang, Mingzhen Ma, Xinyu Zhang, Riping Liu

**Affiliations:** 1 State Key Laboratory of Metastable Materials Science and Technology, Yanshan University, Qinhuangdao 066004, P. R. China; 2 Metallurgy and Materials Science Research Institute, Chulalongkorn University, Bangkok 10330, Thailand,; 3 Research Unit of Advanced Materials for Energy Storage, Chulalongkorn University, Bangkok, Thailand

**Keywords:** BiOI, photocatalytic degradation, p–n heterojunction, ZnO

## Abstract

In this paper, an efficient method to produce a ZnO/BiOI nano-heterojunction is developed by a facile solution method followed by calcination. By tuning the ratio of Zn/Bi, the morphology varies from nanoplates, flowers to nanoparticles. The heterojunction formed between ZnO and BiOI decreases the recombination rate of photogenerated carriers and enhances the photocatalytic activity of ZnO/BiOI composites. The obtained ZnO/BiOI heterostructured nanocomposites exhibit a significant improvement in the photodegradation of rhodamine B under visible light (λ ≥ 420 nm) irradiation as compared to single-phase ZnO and BiOI. A sample with a Zn/Bi ratio of 3:1 showed the highest photocatalytic activity (≈99.3% after 100 min irradiation). The photodegradation tests indicated that the ZnO/BiOI heterostructured nanocomposites not only exhibit remarkably enhanced and sustainable photocatalytic activity, but also show good recyclability. The excellent photocatalytic activity could be attributed to the high separation efficiency of the photoinduced electron–hole pairs as well as the high specific area.

## Introduction

The development of semiconductor photocatalysis has opened a new horizon for environmental pollution remediation and provides a potential solution to the global energy problem given the abundance and sustainability of solar energy. The incredible potential of these materials, which has attracted great enthusiasm from researchers in the past a few decades, renders them strong candidates for a variety of applications that range from photocatalytic water splitting [1–2], organic pollution degradation [3–6], nitrogen fixation [7–8], to solar fuel production [9]. The metal oxides, such as CdO [10], Al_2_O_3_ [11], and CuO [12–13], has attracted a lot of interest in photocatalytic applications. And among all of these metal oxides, it is titanium dioxide and zinc oxide (TiO_2_ and ZnO) that are the most widely exploited by taking advantage of their long-term stability and environmental non-toxicity, in addition to providing a low-cost alternative to the n-type semiconductor photocatalysts [14–16]. However, further application of these metal oxides is usually significantly limited by the poor utilization of visible light, which accounts for 45% of the solar radiation spectrum, because of their wide band gap. Some effort must be paid to extend their working spectrum efficiently.

As is known to all, it is metal orbitals which contribute to the conduction band (CB) of the metal-containing semiconductor’s band gap. Bismuth(III)-containing semiconductors (such as bismuth oxide [17], bismuth vanadate [18–19], bismuth tungstate [20], bismuth perovskite [21], bismuth molybdate [22], etc.) have been extensively researched as a broad hybrid orbital composed of Bi 6s in the field of photocatalysis. Recently, bismuth oxyhalides have been proposed as a new kind of narrow band gap p-type semiconductor photocatalyst material whose general formula is BiOX, where X represents Cl, Br or I. They crystallize in the tetragonal matlockite structure with space group P4/NMMS. In the crystal structure of all these ternary compounds, [Bi_2_O_2_]^2+^ slabs are interweaved with double layers of halide atoms [23]. Calculated and experimental evidence has proven that the band gap of BiOX materials decreases with increasing ionic radii of X (from Cl to I) [24–25]. Hence, BiOI, with a band gap around 1.8 eV, has a strong response to visible light. Though widely investigated, BiOI suffers from its high recombination rate of photoinduced electron–holes and low charge transfer ability. Therefore, the strategies to improve the efficiency of this kind of semiconductor photocatalyst are in need.

Heterogeneous processes are becoming a key focus area for the design of novel and multifunctional materials in the field of photocatalysis. To date, unremitting efforts have been devoted to improving the photocatalysis efficiency [26–29]. Furthermore, coupling n-type semiconductor photocatalysts to p-type to form p–n heterojunctions could promote increased photocatalytic activity efficiency. Once the p–n junction has been formed, the inner electric field between the inner surface of two semiconductors will promote the separation efficiency of photoinduced electron–hole pairs [30–31].

Consequently, coupling an n-type metal oxide to a p-type BiOX to form a p–n heterojunction is an effective method to optimize the photocatalytic activity (summarized in Table S1, Supporting Information File 1). By now, several works have been published in this area. In 2009, Zhang et al. reported a low-temperature route to prepare TiO_2_/BiOI photocatalysts which showed higher activity than single-phase BiOI or TiO_2_ and 50% BiOI possessed the best performance [32]. Jiang and co-workers used a chemical bath to produce ZnO/BiOI heterostructures. By tuning the ratio of Zn/Bi, they could rationally control the morphology, constituents and optical properties [33]. In 2014, spin-coating was utilized to prepare ZnO nanowire arrays with tunable band gap, layered BiOBr–BiOI composites onto them. It showed that the BiOBr/BiOI composites played the role of sensitizer in the heterojunctions and were tunable by varying the BiOBr/BiOI ratio [34]. Kuang et al. described the synthesis of p–n heterostructures where p-type BiOI nanoplates decorated on n-type ZnO nanorod arrays which were synthesized through a solvothermal route. The high-contact areas provided by the fast charge transfer channel of BiOI and ZnO create the efficient photocatalyst [35]. Tong et al. [36] reported ZnO-embedded BiOI hybrid nanoflakes fabricated by using Zn_5_(CO_3_)_2_(OH)_6_ ultrathin nanosheets for BiOI deposition followed by calcination. The obtained ZnO-embedded BiOI hybrid nanoflakes show good photocatalytic activity and recyclability. Jiang et al. [37] prepared the BiOI/ZnO photocatalysts by a two-step synthetic method. The improved photocatalytic degradation of phenol could be achieved under simulated solar light irradiation.

Herein, we report a new, facile method to produce the ZnO/BiOI heterojunction by a solution method followed by calcination. Using a milder precipitant NH_4_HCO_3_ can homogenize the constituent in a relatively smaller range of pH. By tuning the ratio of Zn/Bi, the morphology, which also plays a vital role in photocatalytic dye degradation, varied from nanoplates, to flowers to nanoparticles. The obtained ZnO/BiOI heterostructured nanocomposites showed a significant improvement in the degradation of rhodamine B under visible light (λ ≥ 420 nm) irradiation as compared to ZnO and BiOI. The obtained catalysts with a Zn/Bi ratio of 3:1 exhibited the highest photocatalytic activity. The band gap structure and charge transfer properties are investigated by XPS in detail. With the addition of different kinds of sacrificial agents, the possible ZnO/BiOI heterojunction mechanism of photodegradation is preliminarily discussed.

## Results and Discussion

### Structure and morphology characterization

**Figure 1:**
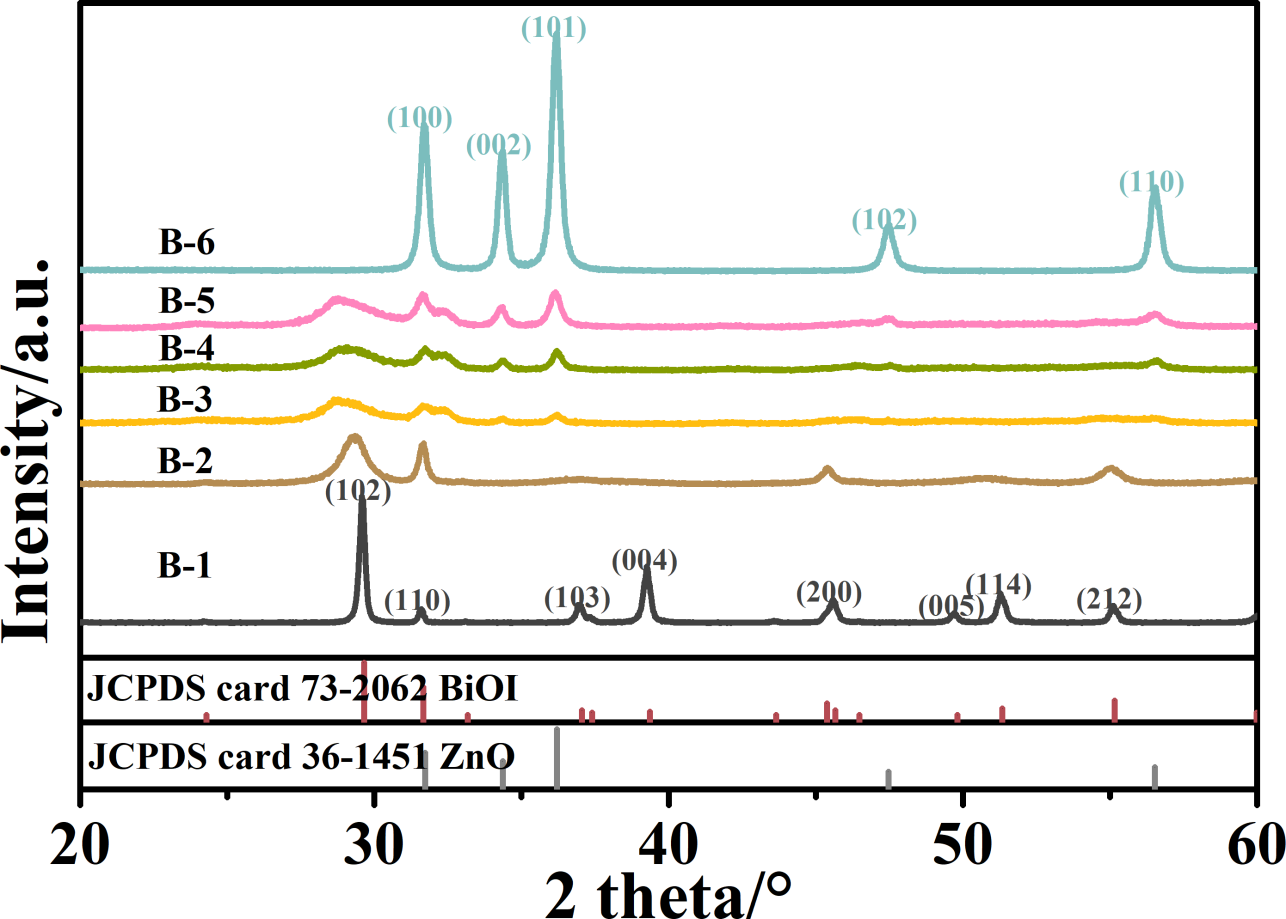
The X-ray diffraction patterns of all of the as-synthesized samples with different Bi/Zn molar ratios (with reference ZnO and BiOI XRD patterns as comparison). All peaks are in good agreement with the tetragonal BiOI and/or the hexagonal ZnO phases.

The composition of as-prepared ZnO/BiOI heterojunction nanocomposites was examined by X-ray diffraction (XRD) analysis as shown in the Figure 1. It is obvious that all the XRD patterns are in good agreement with the tetragonal BiOI (JCPDS card 73-2062) and the hexagonal ZnO (JCPDS card 36-1451) phases. Moreover, for ZnO/BiOI nanocomposites, the diffraction peaks of BiOI tend to decrease while the ZnO diffraction peaks gradually increase, which is consistent with the composite variation from B-2 to B-5. It is worth noting that the (102) diffraction peaks corresponding to BiOI shift to lower angles as well as become weaker and broader, while the Zn/Bi ratio increases (from B-2 to B-5). To give more details on ZnO’s impact on the crystallization of BiOI, the (102) diffraction peak is chosen to roughly estimate the average BiOI crystal size of samples B-2 through B-5 by the Scherrer equation (Table S2, Supporting Information File 1). The estimates show that the average crystal size of BiOI decreases with increasing ZnO, which indicates that the existence of ZnO may impede the crystallization and lead to the exfoliation of BiOI [33]. Y. Tong [36] also reported the broadening and shifting of the (102) diffraction peak of ZnO/BiOI composite samples, which is never mentioned in the previous studies without post-annealing treatment [33]. It is reasonable to believe that the bonding between ZnO and BiOI is stronger after the calcination and can be beneficial to the charge transfer in the photocatalytic activity process.

**Figure 2:**
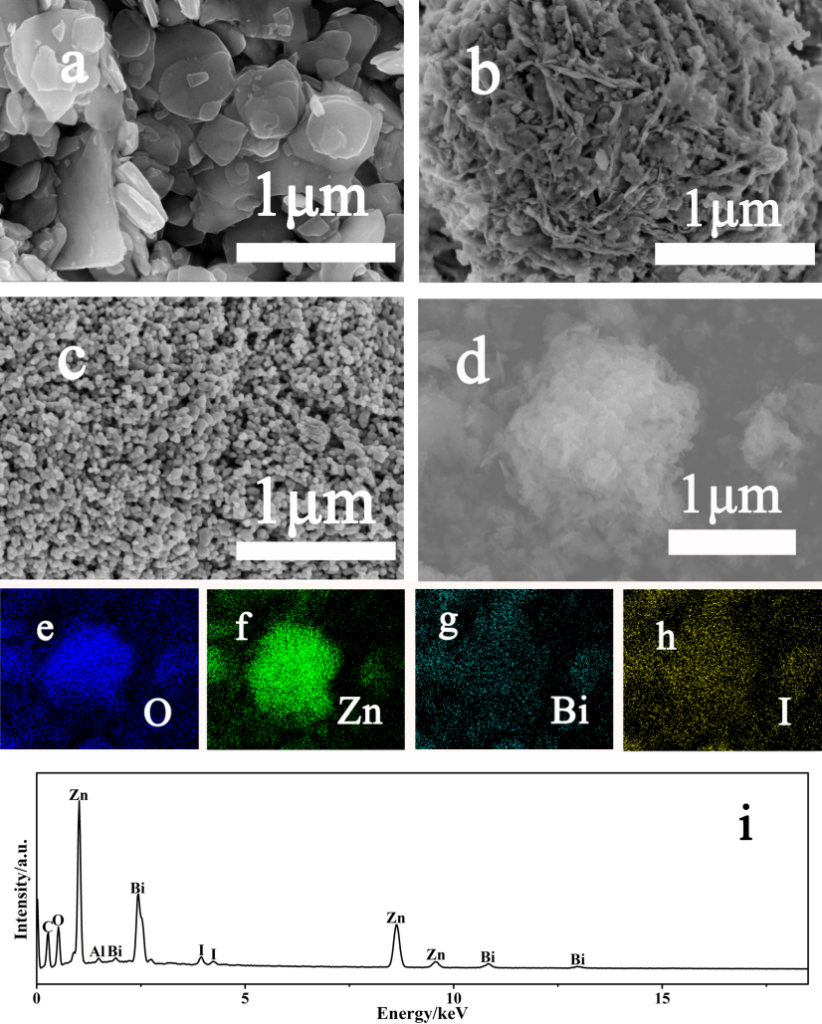
SEM images of sample B-1 (a), B-4 (b), B-6 (c), and B-4, (d). Energy-dispersive spectroscopy (EDS) elemental mapping of (e) O, (f) Bi, (g) Zn, (h) I, and (i) EDS of sample B-4.

Figure 2a–c shows the morphology of samples B-1, B-4 and B-6 from scanning electron microscopy (SEM). In Figure 2a, pure BiOI shows a smooth plate-like shape with thickness varying from 100 to 500 nm and particle diameter of around 2 µm. For sample B-2 (Figure S1a, Supporting Information File 1), either the thickness or the size of the particle has remarkably decreased. The particle size further diminishes with the addition of ZnO (B-3, Figure S1b, Supporting Information File 1). When the Zn/Bi ratio becomes 1:3 (Figure 2b), the exfoliated BiOI ultrathin layers assemble together to spontaneously form a hierarchical microsphere to reduce the surface energy. Coupling the elemental distribution displayed by the energy-dispersive spectroscopy (EDS) mapping (Figure 2d–h) and EDS (Figure 2i), it is clear to see that in this 3D nanoflower structure of B-4 sample the ZnO nanoparticles are uniformly embedded on the architecture built up by erect BiOI layers. However, when the Bi/Zn ratio turns to 1:4 (B-5, Figure S1c, Supporting Information File 1), the morphology of the sample has greatly deviated from that of samples B-1–B4, which is characterized as irregular agglomerate nanoparticles. This indicates that a large amount of ZnO particles take the place of BiOI nanolayers. As pure ZnO, sample B-6 (Figure 2c) presents nanospheres with a highly smooth surface, which is possibly caused by calcination [38].

**Figure 3:**
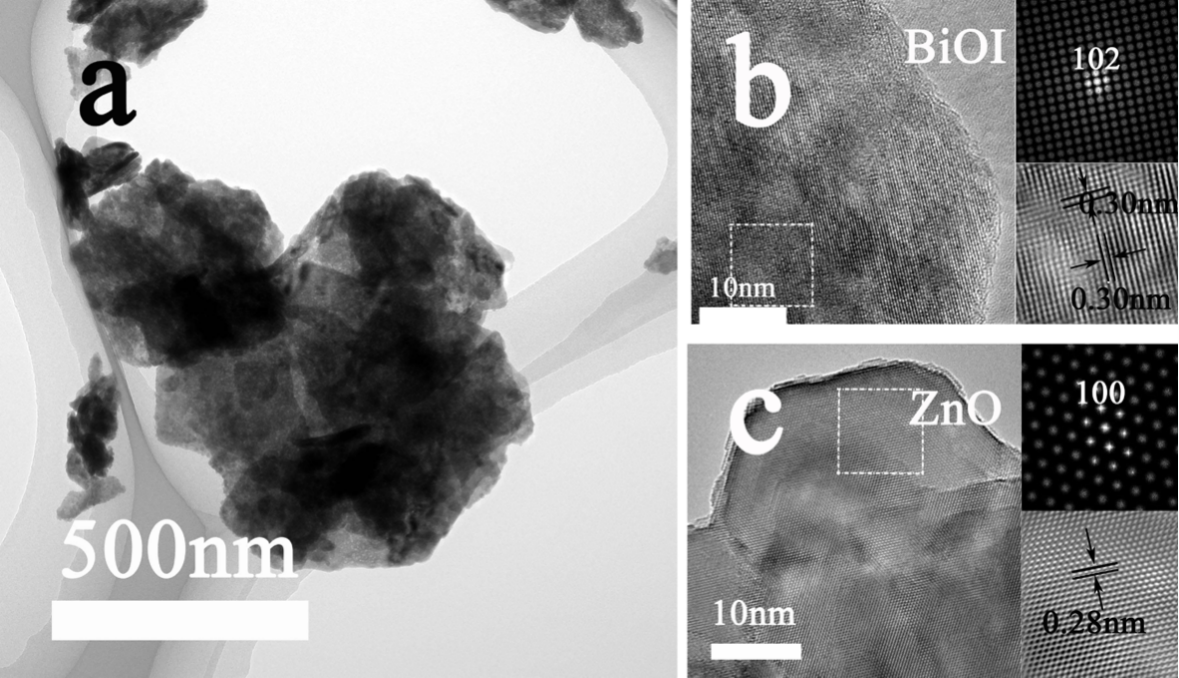
(a) TEM image of sample B-3 and high-resolution images further confirm that a heterostructured ZnO (b) and BiOI (c) nanocomposite is obtained.

In order to make further investigations on the ZnO/BiOI heterostructure, TEM and HRTEM analysis were applied. Figure S2a, Supporting Information File 1, shows the transmission electron microscopy (TEM) image of pure BiOI. It is clear to see that the as-synthesized BiOI forms into irregular nanoplates with smooth edges. Figure S2b, Supporting Information File 1, shows the TEM image of sample B-3 whose Bi/Zn molar ratio equals to 1:2. The morphology of the sample particle displayed here highly agrees with the results of the SEM image, where ZnO aggregates are decorated on the exfoliated BiOI nanolayers. The selected area electron diffraction (SAED) pattern of BiOI (Figure S2c, Supporting Information File 1) reveals that the sample is highly crystalline, as proved by representative SAED pattern of single crystalline with bright spots. The diffraction spots corresponding to (110) and (200) planes are indexed to the {001} facets of tetragonal phase BiOI, which is consistent with the XRD results [39].The concentric circles in the SAED pattern of B-3 (Figure S2d, Supporting Information File 1) illustrate the polycrystalline nature of sample B-3 and the coexistence of BiOI and ZnO. Two bright inner rings, which are indexed to BiOI (106) and ZnO (004) facets, indicate the high crystalline degree and echo the previous XRD study to a great extent. The TEM and HRTEM images on the different edges of sample B-3 are displayed in Figure 3a–c, where lattice fringe spacings are 0.30 and 0.28 nm, respectively, matching well with the interplanar distances of the (102) plane in BiOI and the (100) plane in ZnO. These results further validate the formation of heterostructured nanocomposites of ZnO and BiOI.

The structural characteristics of the as-prepared ZnO/BiOI composites were revealed by nitrogen adsorption–desorption isotherms shown in Figure S3, Supporting Information File 1. The isotherm of sample B-4, with a unique hysteresis loop and a classical two capillary condensation step, undoubtedly corresponds to a type IV isotherm according to the Brunauer–Deming–Derning–Teller (BDDT) classification, indicating the mesoporous nature of sample B-4 [40]. The isotherm has a loop with the shape of H3, which demonstrates that the pores in the sample are mainly made up of slit-like cracks attributed to the aggregation of ultrathin BiOI layers and is consistent to the SEM image of the sample B-4 in Figure 2b. The Brunauer–Emmett–Teller (BET) analysis also illustrated that the specific area (listed in Table S2, Supporting Information File 1) of the B-4 sample is 20.83 cm^2^/g, which is significantly improved from the pure BiOI (5.23 cm^2^/g) and ZnO (16 cm^2^/g) materials. This may lead ZnO/BiOI to have more active bonding sites than pure BiOI and ZnO, which is beneficial for the photodegradation activity.

### Chemical state analysis

X-ray photoelectron spectroscopy (XPS) was carried out for the chemical composition and valence state analysis of various species. The foreign impurity C at 284.6 eV is used to calibrate peak positions in all the XPS results. High-resolution narrow-scan spectra of O, Bi, Zn and I are also conducted for the study of surface chemical state in detail.

**Figure 4:**
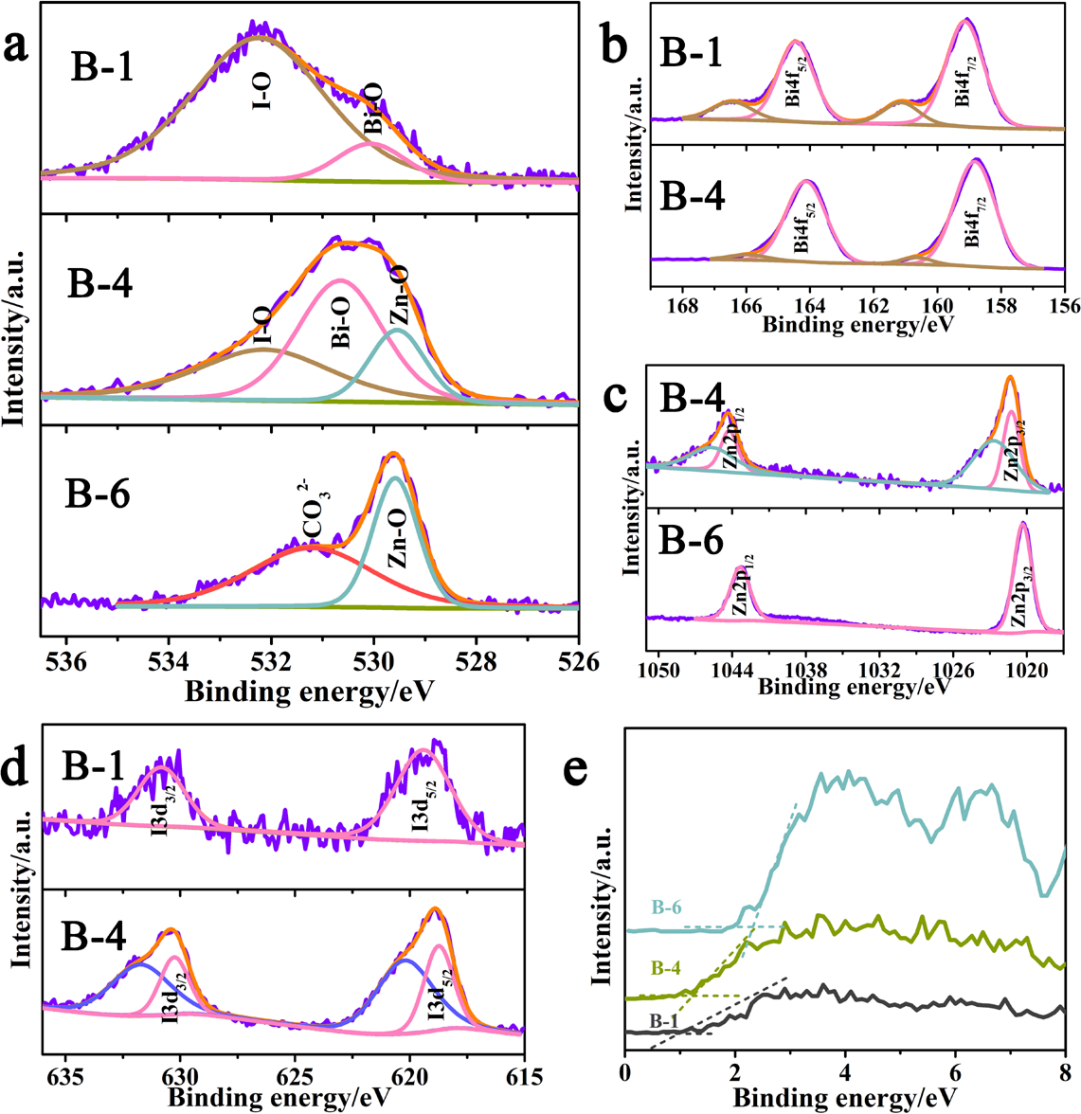
The high-resolution XPS spectrum for: (a) O 1s core energy of B-1, B-4 and B-6; (b) Bi 4f of B-1 and B-4 samples; (c) Zn 2p of B-4 and B-6 samples; (d) I 3d of B-1 and B-4 samples; and (e) XPS valence band spectrum of samples B-1, B-4 and B-6.

The O 1s spectra of B-1, B-4 and B-6 are recorded in Figure 4a. The spectrum of O 1s for pure BiOI can be fitted with two peaks that are centered at a binding energy of 532 eV and 530 eV. The dominant peak at 532 eV is attributed to I–O bonds in BiOI, and the peak located at 530 eV is assigned to oxygen species of lattice oxygen (O^2−^) [22,41–42] which is related to Bi–O in [Bi_2_O_2_] slabs of BiOI. In the typical ZnO/BiOI heterostructured composite B-4, the high-resolution spectrum of O 1s can be deconvoluted into three components. The I–O peak which is centered at around 532.3 eV is the same as pure BiOI, while the peak that belongs to lattice oxygen could be further deconvoluted into two peaks located at 530.5 eV and 529.5 eV, respectively. It is noticeable that in the previous study, the peak with high energy is ascribed to Zn–O bonds whereas the lower energy peak is attributed to Bi–O bonds [32–33]. However, in the O 1s spectra of the B-6 sample (pure ZnO), the O lattice peak is at 529.6 eV, corresponding to the Zn–O bonds in ZnO [43]. Combining the O 1s high-resolution XPS results of B-1, the peak in the B-4 sample with higher energy at 530.5 eV should be Bi–O [44] and the peak at a lower energy of 529.5 eV should be Zn–O. The subtle deviation between the peak positions in the O 1s spectra of the ZnO/BiOI sample and pure ZnO and BiOI confirms the formation of the heterojunction and the charge transfer from Bi–O bonds to Zn–O bonds in the nanocomposite sample [45].

The high-resolution scan of Bi 4f is displayed in Figure 4b. Both pure BiOI and the nanocomposite sample consist of a doublet which can be well-assigned to Bi 4f_5/2_ at 164.5 eV and Bi 4f_3/2_ at 159.2 eV with an energy difference of 5.3 eV [39,46], revealing that +3 is the main valence state of the Bi element in B-1 and the nanocomposite sample [17]. A pair of satellite peaks centered at about 1.8 eV higher than the main peaks can be identified as Bi^5+^ defects and agreed well with the reported values [30,32]. As shown in Figure 4c, there are two separate peaks with a difference of 23 eV in Zn 2p region of B-4 and B-6 samples. They are characteristics of Zn 2p_1/2_ at 1043 eV and Zn 2p_3/2_ at 1020 eV ascribed to the Zn^2+^ state [47]. In the Zn 2p core level of B-4, a pair of satellite peaks are observed with binding energy of 1045.65 and 1022.65 eV, which are probably caused by the change of chemical state of Zn attributed to ZnO compositing to BiOI. Interestingly, in Figure 4b,c, the Bi 4f and Zn 2p peaks of the nanocomposite sample are more or less shifted to higher energy as compared with the single phase, but maintain the spin-orbit splitting at about 5.3 and 23 eV, respectively. This indicates that the main states of Bi and Zn are +3 and +2 in the ZnO/BiOI heterostructured composite [48].

Figure 4d exhibits the high-resolution results of I 3d XPS spectra. Two peaks located at 619.4 eV and 630.8 eV can be assigned to I 3d_5/2_ and I 3d_3/2_ according to the previous works for pure BiOI [46]. After coupling with ZnO, however, the satellite peaks appeared in the B-4 sample, demonstrating the chemistry state of I is changed. This probably resulted from the partial substitution of I for oxidic sites in the ZnO framework [49]. The density of electronic state (DOS) of the valence band was also characterized by XPS, yielding information pertaining to the bonding properties of different samples, as displayed in Figure 4e. The B-1 and B-6 samples show typical valence band DOS characteristics of BiOI and ZnO, with edge cutoff values of 0.96 eV and 2.27 eV, respectively [50]. For the B-4 sample, the edge cutoff value is about 1.22 eV. The change in the state, along with the difference in the shape from pure BiOI and ZnO, is probably due to the band bending attributed to the formation of ZnO–BiOI heterojunctions, as its Bi/Zn molar ratio is 1:3.

### Optical properties

To investigate the photo-induced charge carrier separation efficiency, photoluminescence (PL) emission spectra was carried out and the results are displayed in Figure S4, Supporting Information File 1. Generally speaking, stronger PL intensity indicates a higher photoinduced hole–electron recombination rate, which is not in the photocatalysts favor. As can be clearly seen, B-1 and B-6 exhibited an extremely strong intensity, demonstrating the lower charge separation efficiency of pure BiOI and ZnO. The intensity greatly decreased as the BiOI/ZnO ratio equals 1:3, revealing that the formation of the ZnO/BiOI heterostructured composite is beneficial for improving the charge separation ability.

**Figure 5:**
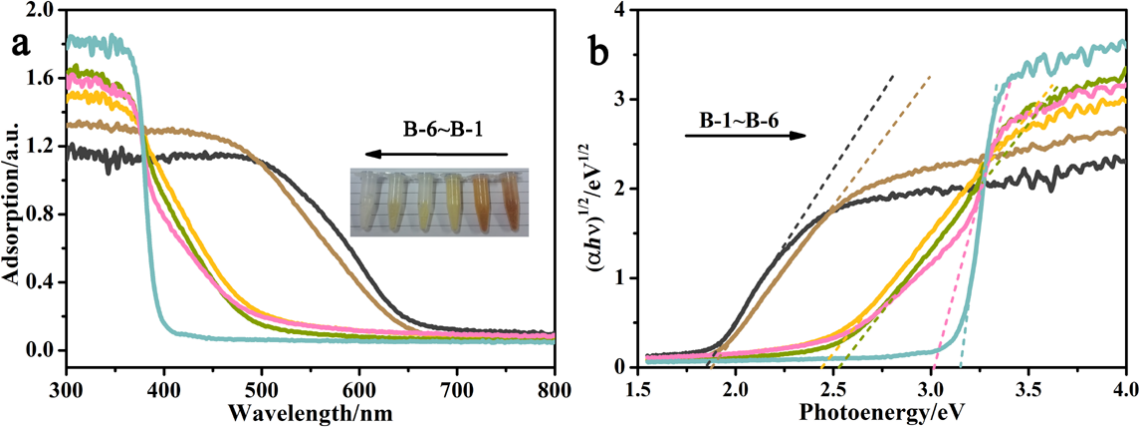
Optical properties of the as-prepared samples (a) UV–vis DRS spectra where the inset shows the physical color of the samples. (b) The plot of (α*h*ν)^2^ vs photon energy (*hν*).

The light adsorption ability and the band gap structure are some of the decisive factors for photocatalysis. Therefore, the UV–vis diffuse reflection spectra (UV-DRS) is worthy of study to explore their optical properties. The UV-DRS results are shown in Figure 5a (inset is the colors of the B-1–B-6 samples which vary from orange-red to white) for the wavelength range 300–800 nm. Pure ZnO (B-6) shows a cut-off at about 400 nm, and pure BiOI (B-1) has an absorbance edge at about 660 nm. The other samples with the Bi/Zn molar ratio varying from 1:1 to 1:4 displayed a gradually decreasing adsorption edge from the visible light range to the ultraviolet region. To determine the conduction positions, the Tauc relation is utilized to estimate the optical band gap. According to the following relational expression proposed by Tauc, Davis and Mott:

[1]
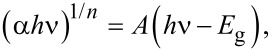


where *h* is Plank’s constant, *ν* is the frequency of vibration, α is the adsorption coefficient, *E*_g_ is the optical band gap and *A* is a proportionality constant [51–52]. The value of the constant *n* denotes the characteristics in the transition of a semiconductor. For the case that both ZnO and BiOI indirectly allow the transition, *n* equals 2. The plots that depict the value of the photoenergy against (α*hν*)^1/2^ and respective tangents, based on the relation mentioned above, is shown in Figure 5b. The points of the intersections of the tangents to the *x*-axis become the approximate values of the band gap energy of the as-prepared samples, as listed in Table 1. This further illustrates that the band gaps of the samples improve with the increase of the amount of ZnO. It is particularly noteworthy that the band gap of as-synthesized ZnO is 3.16 eV, which is smaller than the band gap of conventional bulk ZnO, probably a result of the quantum confinement effect in the nanometer-sized ZnO particles.

**Table 1:** The optical band gap, degree of degradation for 100 min and the reaction rate coefficient of the as-prepared samples as compared to the reference samples (blanks).

Sample	B-1	B-2	B-3	B-4	B-5	B-6	blank

optical band gap (eV)	1.85	1.90	2.46	2.52	2.96	3.16	–
degradation degree after 100 min (%)	74.0	96.9	96.1	99.3	92.7	21.2	1.6
*K* (×10^−4^ min^−1^)	129.5	303.1	297	462	266.1	23.5	1.17

### Photocatalytic degradation

**Figure 6:**
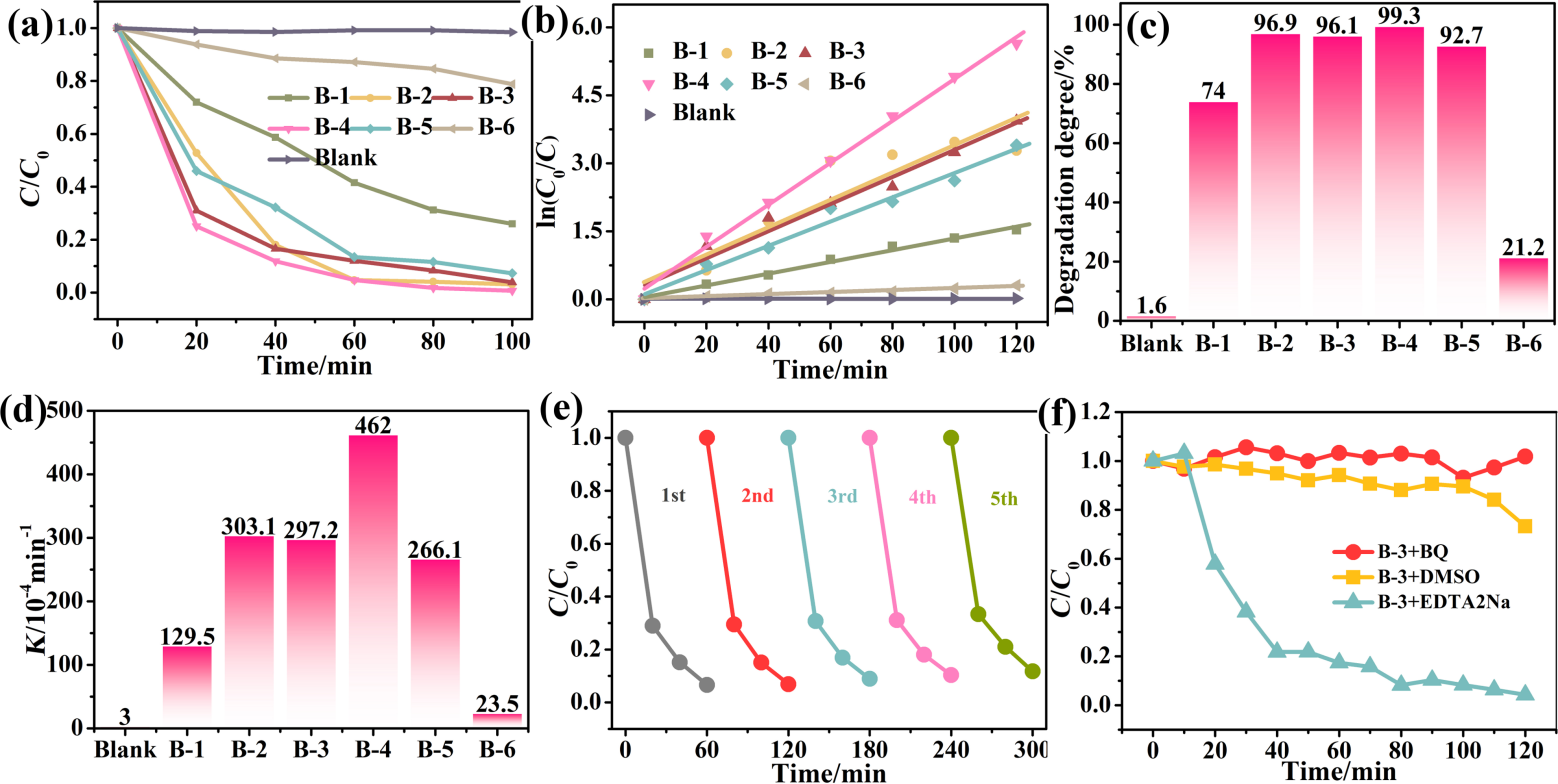
Photocatalytic activity under visible light illumination. (a) The rhodamine B (RhB) solution degradation of the as-prepared samples with sample with no catalyst and P25 as comparison. (b) The linear fitting of the pseudo-first kinetic model. (c) Photodegradation degree within 100 min of the as-synthesized sample. (d) Photocatalytic rate of all the samples. (e) Recycling tests for the B-4 sample whose Bi/Zn molar ratio is 13. (f) The effect of sacrificial agents on the RhB photodegradation of the B-3 sample (Bi/Zn 1:2).

To test the as-synthesized samples’ ability to remove organic pollutants from waste water, the photodegradation of RhB was carried out under illumination by visible light from a 300 W Xe lamp with a 420 nm cut-off filter. In order to make a valid comparison of the degradation effectiveness of the as-prepared samples, the initial adsorption in the dark is ruled out. The adsorption–desorption equilibrium is displayed in Figure S5, Supporting Information File 1. For a comprehensive comparison, the blank group was conducted under the same conditions. The results can be intuitively observed in Figure 6a, where *C*_0_ represents the initial dye concentration and *C* is the concentration after the photocatalytic testing. The results show that the degradation performance of ZnO and BiOI are quite unsatisfactory, while no concentration difference is visible without the catalyst, demonstrating the self-degradation of RhB is negligible. All of the ZnO–BiOI heterostructured nanocomposites exhibit remarkably improved photocatalytic activity in terms of both the efficiency and the degradation degree for 100 min (Table 1). With reducing the Bi/Zn molar ratio, the degradation degree for 100 min of the sample becomes higher. After the most optimal point has been reached by B-4 sample (with the Bi/Zn ratio of 1:3), the performance of sample B-5 drops off, which is likely to be triggered by the reduction of the heterojunction with further increasing amount of ZnO.

To exploit the kinetic process of the photocatalytic reaction, the RhB degradation data can be fitted to the pseudo-first order reaction kinetic model ln(*C*_0_/*C*) = *Kt* in Figure 6b, where *K* is the reaction rate coefficient (Table 1). The photodegradation degree within 100 min and the photocatalytic rates of the as-synthesized samples compared with blank controls are shown in Figure 6c,d. Among all the samples and the control groups, B-4, with the highest degradation degree, also displays the highest reaction rate coefficient, confirming that the Bi/Zn molar ratio of 1:3 is the most optimal sample for photocatalytic performance. Other very critical criteria of the photocatalyst for practical application are stability and reusability. Figure 6e presents the recycling test results of the B-4 sample. After being circularly used for five times, the degradation degree still remains at 95% photocatalytic activity compared to the result of the first cycle, indicating the stability and the reuse ability are considerably high.

It is known that the degradation of organic dye is realized through redox reactions, which are mainly dependent on the oxidative and/or reductive species. To obtain a clear consensus of the reactive species and to further explore the photocatalytic degradation mechanism in detail, an examination was carried out by adding different sacrificial agents at a calculated amount. Benzoquinone (BQ), dimethyl sulfoxide (DMSO) and ethylene diamine tetraacetic acid sodium (EDTA sodium) were adopted as scavengers of O_2_^•−^ [53], ^•^OH [54] radicals and holes [55], respectively. From the results displayed in Figure 6f, it is obvious that while the addition of BQ significantly inhibits the photocatalytic activity, DMSO has less effect on the degradation degree. EDTA sodium has little impact on the photodegradation of RhB solution under visible light. Thus, although produced in the photocatalytic processes, the photoinduced holes do not play an important role in the degradation reaction. Therefore, the decomposition of RhB solution is mainly attributed to the oxidation reaction of O_2_^•−^ and the ^•^OH radicals. Based on the results of the experiments, the probable processes of the mechanism can be proposed as follows:

[2]
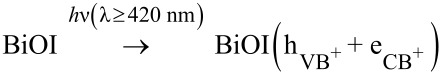


[3]
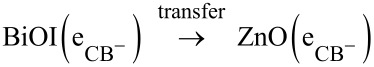


[4]
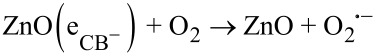


[5]
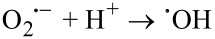


[6]
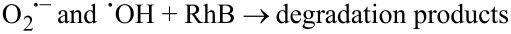


### Photocatalytic mechanism

**Figure 7:**
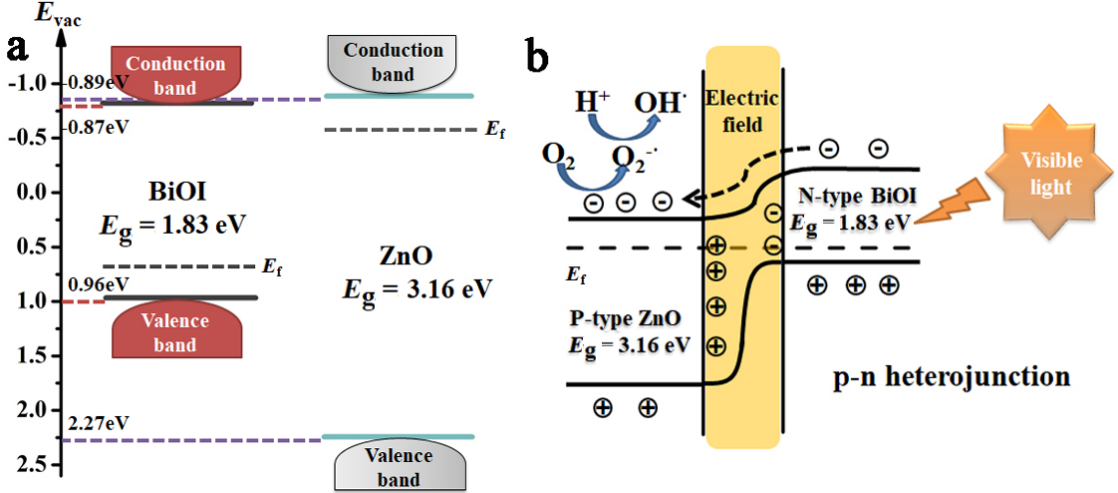
Schematic band energy diagram of BiOI and ZnO before (a) and after (b) interfacial contact.

On the basis of above results, it is firmly believed that the photocatalytic degradation ability of ZnO/BiOI nanocomposites was greatly improved due to the p–n heterojunction structure between ZnO and BiOI. Further insight into the mechanism is illustrated as follow. Figure 7a shows the simplified energy band structure of BiOI and ZnO. The valence band maximum energies of BiOI and ZnO as obtained in XPS measurements are 0.96 and 2.27 eV, and the band gaps calculated from UV–vis spectra are 1.83 eV for BiOI and 3.16 eV for ZnO. Thus, the conduction band minimum energy should occur at –0.87 eV and –0.89 eV for BiOI and ZnO, respectively. While BiOI possesses a p-type semiconductor material nature whose Femi level is located not far from the valence band, ZnO is a typical n-type material with the Femi level near the conduction band. When BiOI was hybridized by ZnO, the construction of a p–n heterojunction realigned the Femi levels of the two materials to the same energy to form an equilibrium state, hence the band bending and the electric field are created consequently [56] and photoinduced electrons are transferred to the conduction band of ZnO and leave the holes in the valence band of BiOI (Figure 7b). This indicates that the separation efficiency of the ZnO/BiOI nanocomposite is remarkably improved, thus the photocatalytic degradation ability of the heterostructure sample is better than the single phase.

## Conclusion

ZnO–BiOI heterostructured nanocomposites were successfully synthesized via a facile solution method followed by calcination, resulting in different morphology, optical properties and band gap structures by tuning the Bi/Zn molar ratio to 1:0, 1:1, 1:2, 1:3, 1:4 to 0:1. The results of the photocatalytic degradation of rhodamine B under visible-light irradiation demonstrate that the as-synthesized ZnO/BiOI heterostructured nanocomposites exhibit the superior photocatalytic ability as compared to pure BiOI and ZnO, which could be attributed to the synergistic effects of higher specific area and the enhanced separation efficiency of photoinduced electron–hole pairs.

## Experimental

### Chemicals

Zinc acetate (Zn(CH_3_COO)_2_·2H_2_O), bismuth nitrate (Bi(NO_3_)_3_·5H_2_O), potassium iodine (KI), ammonium hydrogen carbonate (NH_4_HCO_3_), rhodamine B (RhB), benzoquinone (BQ), dimethyl sulfoxide (DMSO) and ethylenediamine teraacetic acid sodium (EDTA sodium) were directly used without any further purification.

### Synthesis of ZnO–BiOI nanocomposites

Generally, 0.05 mol NH_4_HCO_3_ were added into 50 mL DI water to form solution A, and 0.025 mol Zn(CH_3_COO)_2_·2H_2_O was dissolved in 25 mL DI water which was denoted as solution B. After the 30 min of stirring, solution B was slowly poured into solution A with continuous stirring for about 1 h. Subsequently, the required amount of Bi(NO_3_)_3_·5H_2_O (Bi/Zn molar ratios which range from 1:0, 1:1, 1:2, 1:3, 1:4 to 0:1 denoted as B-1, B-2, B-3, B-4, B-5 and B-6, respectively), dissolved in acetic acid, was added dropwise to the above-mentioned mixture of solution A and B. KI at an appropriate amount was added as the iodine source. The solution was stirred at 80 °C for 2 h. The resulting samples were centrifuged, washed thoroughly with distilled water, and then dried at 65 °C for 12 h. Finally, the heterostructured ZnO/BiOI nanocomposites were obtained after calcination at 300 °C for 2 h in air. For comparison, the pure ZnO was prepared by the same procedure but without using the BiOI precursor, and the preparation of pure BiOI was also fabricated without using ZnO precursor, based on the method described by Yu et al. [57].

### Characterization

X-ray diffraction (XRD) was conducted on a Rigaku A/MAX-Rb system operated at 40 kV and 100 mA with Cu Kα radiation, with a scanning rate of 4°/min in the range of from 10 to 90°. A Hitachi S4800 scanning electron microscope (SEM) was used to investigate the morphology with an accelerating voltage of 15 kV. The elemental analysis was also performed on an energy-dispersive spectrometer (EDS) with the same instrument. Transmission electron microscopy (TEM) was employed to observe the microstructure of the as-synthesized ZnO/BiOI heterojunction nanocomposites under the operating voltage of 120 kV on a JEOL-ARM200F device. The surface area and the pore distribution were tested by the nitrogen adsorption–desorption isotherms recorded on a ASAP 2020 HD88 using the Brunauer–Emmett–Teller (BET) method at liquid nitrogen temperature 77 K.

The optical absorption properties of the samples were measured by UV–vis spectra on a Hitachi U-4100 spectrophotometer equipped with an integrating sphere at room temperature in the region of 200–800 nm. The photoluminence (PL) spectra were recorded by a Horiba Floruomax-4_1188D-4513-FM instrument with an emission wavelength of 320 nm. X-ray photoelectron spectroscopy (XPS) measurements were performed at 150 W with a monochromatic Al Kα (1486.6 eV) X-ray source on an ESCALAB 250 instrument. The binding energy correction was carried out using the C 1s peak of 284.6 eV as reference.

### Photocatalytic tests

The photocatalytic activity of ZnO–BiOI nanocomposites was evaluated by degradation of rhodamine B (RhB) under irradiation by a 300 W Xe lamp with a 420 nm cut-off filter at ambient temperature. 0.1 g of sample was added to 100 mL of 10 mg/L RhB DI water solution in the reactor. To achieve the balance of adsorption–desorption in the uniform suspension, the system was sonicated and magnetic stirred for 30 min in the dark. After the irradiation was introduced into the system, the concentration of RhB was periodically detected by the UV–vis spectrum to determine the photodegradation degree with continuous stirring. For reactive species investigation, specific scavengers were introduced into the reaction system by following the same procedures as described in the photodegradation experiments.

## Supporting Information

File 1 Additional experimental information.
