# Supplementary material for: Facile synthesis of a ZnO–BiOI p–n nano-heterojunction with excellent visible-light photocatalytic activity
Source: Beilstein J Nanotechnol. 2018 Mar 5;9:789–800. doi: 10.3762/bjnano.9.72 (PMC5852526; doi:10.3762/bjnano.9.72)
Supplement: File 1 — Additional experimental information. [file Beilstein_J_Nanotechnol-09-789-s001.pdf]

Supporting Information

for

Facile synthesis of a ZnO–BiOI p–n nano-heterojunction with excellent visible-light photocatalytic activity

Mengyuan Zhang[1], Jiaqian Qin[2,3]*, Pengfei Yu[1], Bing Zhang[1], Mingzhen Ma[1], Xinyu Zhang[1,]*, and Riping Liu[1]

Address: [1]State Key Laboratory of Metastable Materials Science and Technology, Yanshan University, Qinhuangdao 066004, P. R. China, [2]Metallurgy and Materials Science Research Institute, Chulalongkorn University, Bangkok 10330, Thailand, and [3]Research Unit of Advanced Materials for Energy Storage, Chulalongkorn University, Bangkok, Thailand

Email: Jiaqian Qin - jiaqian.q@chula.ac.th; Xinyu Zhang - xyzhang@ysu.edu.cn

*Corresponding Author

Additional experimental information

Table S1: Comparison of ZnO/TiO_2-BiOI composites prepared by different methods

Samples	Synthetic method	Testing method	Test result	Ref.
**ZnO-BiOI nanoheterojunction**	Solution method followed by calcination.	Photocatalytic degradation of RhB solution under 300 W Xe lamp with a 420 nm cutoff filter.	99.3% of RhB was degraded after 100 min irradiation.	This report
**TiO2-BiOI heterostructure**	Soft chemical method	Photocatalytic degradation of MO solution under 500 W halogen-tungsten lamp with a 420 nm cutoff filter.	95% of MO was degraded after 2h irradiation.	[1]
**ZnO-BiOI heterostructure**	Chemical bath method	Photocatalytic degradation of MO solution under 500W halogen-tungsten lamp with a 420 nm cutoff filter.	78% of MO was degraded after 4h irradiation.	[2]
**BiOBr-BiOI/ZnO nanowires heterostructure**	Spine-coating	Photocurrent under the 300 W Xe lamp with a 420 nm filter.	4.8 mA/cm^2	[3]
**BiOI nanoplate- ZnO nanorod p-n heterojunction**	Electrodeposition followed by solvolthermal process	Photoelecrocatalytic degradation of CR under 300 W Xe lamp with a 420 nm filter.	93.66% of CR was degraded after 2 h irradiation.	[4]
**ZnO embedded into BiOI hybrid nanoflakes**	Solvolthermal method followed by precipitation-deposition in combination with calcination.	Photodegradation of RhB under the irradiation of 300 W Xe lamp with a 420 nm filter.	37.2% of the RhB was degraded after 5 min irradiation.	[5]
**BiOI loaded ZnO nanorods composites**	Solvolthermal method followed by precipitation-deposition	Photodegradation of phenol under the irradiation of 500 W Xe lamp without filter.	99.9% of penol was degraded after 2 h irradiation.	[6]

Table S2: The average crystal size estimated by Scherrer formula and the specific surface area of selected samples.

**Samples**	**Crystal size(nm)**	**Specific surface area (cm^2/g)**
B-1	-	5.23
B-2	31.25	-
B-3	14.8165	-
B-4	14.2949	20.83
B-5	12.5145	-
B-6	-	16

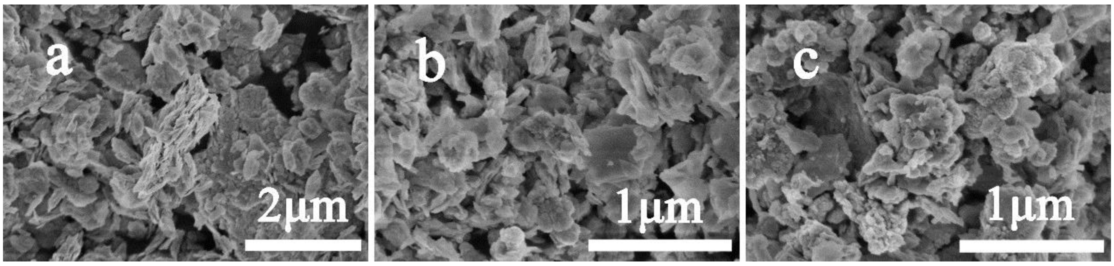

Figure S1: SEM image of a. B-2 (Bi/Zn=1/1), b. B-3 (Bi/Zn=1:2) and c. B-5 (Bi/Zn=1/4). From figure S1 a and b, it is obviously seen that the thickness and size of the particles from B-2 and B-3 samples diminish with the increase of the concentration of ZnO. When the Bi/Zn molar ratio reaches to 1/4, the assembled ZnO nano particles displace of the plate-like BiOI as the major shape of the B-5 sample.

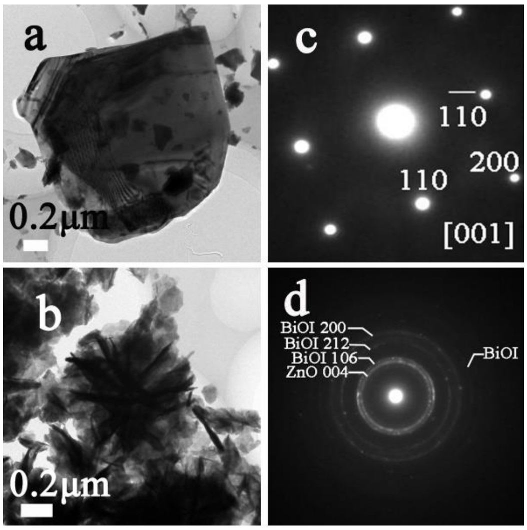

Figure S2: The TEM images: a. pure BiOI, b. B-3 sample and their SAED patterns: c. pure BiOI, d. B-3 sample. The morphology of the samples highly agree with the results of the SEM images. The crystallinity natures of B-1 and B-3 are each revealed by figure S2 c and d, which represent single and poly crystal feature respectively.

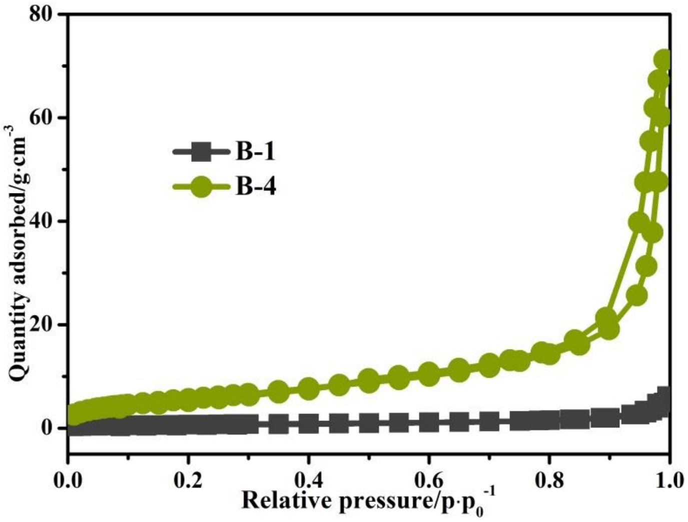

Figure S3: N_2 adsorption-desorption isotherms of pure BiOI and B-4 sample with Bi/Zn molar ratio=1/3. The B-4's shape corresponds to a type IV isotherm, with a loop shape of H3, demonstrating the pores in B-4 are mainly made up of slit-like cracks.

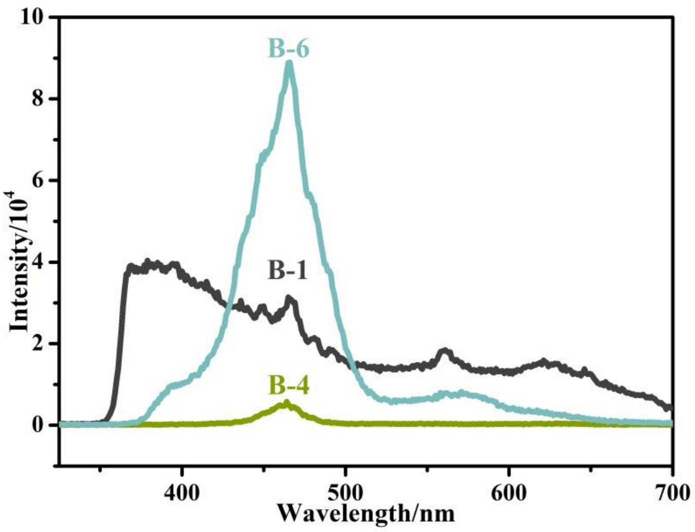

Figure S4: Photoluminescence spectra of B-1, B-4 and B-6 samples. The PL spectrum of B-4 has the lowest intensity comparing with the pure BiOI (B-1) and ZnO(B-6), illustrating the highest photo-induced charge separation efficiency.

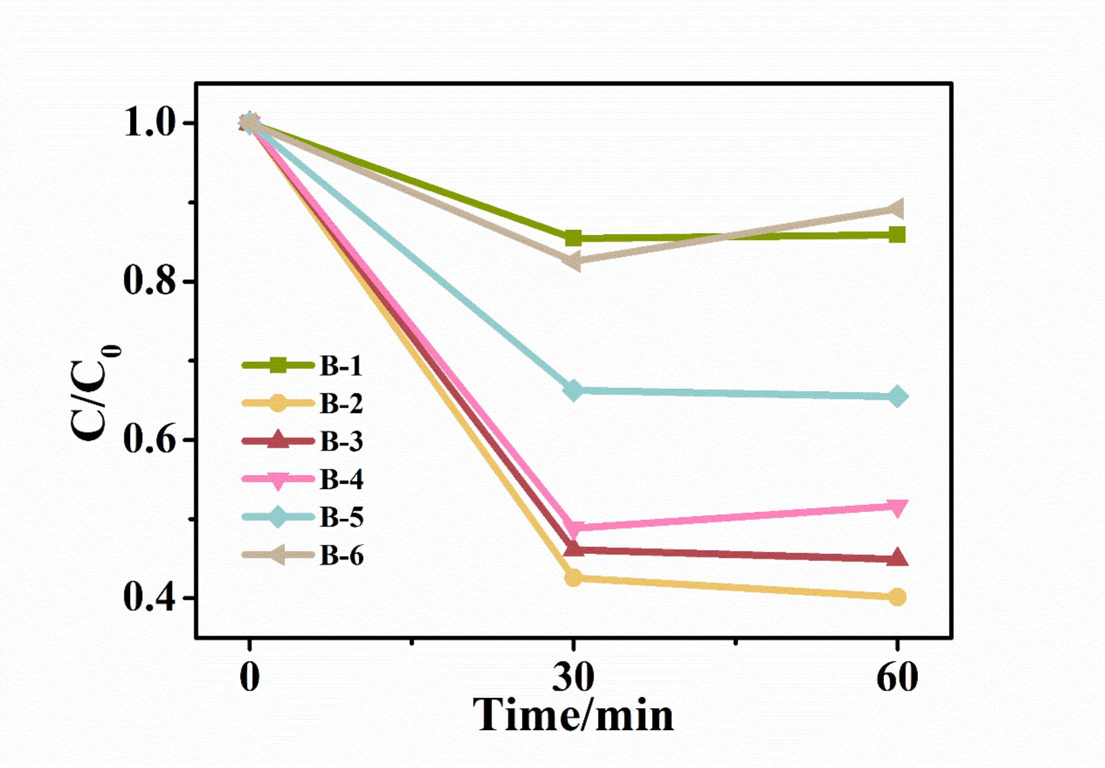

Figure S5: Adsorption desorption equilibrium of different samples in the dark.

References

1 Zhang X., Zhang L. Z., Xie T. F., Wang D. J. *J. Phys. Chem. C*. **2009**,113, 7371-8.

2 Jiang J., Zhang X., Sun P. B., Zhang L. Z. *J. Phys. Chem. C*. **2011**,115, 20555-64.

3 Yu Y. X., Ouyang W. X., Zhang W. D. *J. Solid State Electrochem*. **2014**,18, 1743-50.

4 Kuang P. Y., Ran J. R., Liu Z. Q., Wang H. J., Li N., Su Y. Z., et al. *Chem – Eur J*. **2015**, 21, 15360-8.

5 Tong Y. H., Zheng C., Lang W. J., Wu F., Wu T., Luo W. Q., et al. *Mater Des*. **2017**, 122, 90-101.

6 Jiang J. J., Wang H., Chen X. D., Li S., Xie T. F., Wang D. J., et al. *J. Colloid Intersurface Sci*. **2017**, 494, 130-8.
